# A Sulfur-Crosslinked Biopolymeric Matrix for Controlled Urea Release Enhances Maize Growth and Reduces Nitrogen Losses

**DOI:** 10.3390/ijms27093863

**Published:** 2026-04-27

**Authors:** Ana Farioli, Pablo Cavallo, Diego Acevedo, Edith Yslas

**Affiliations:** 1 Instituto de Investigaciones en Tecnologías Energéticas y Materiales Avanzados, IITEMA, (CONICET-UNRC) Departemento de Tecnología Química, Facultad de Ingeniería, Universidad Nacional de Río Cuarto, Río Cuarto X5800, Argentina; afarioli@exa.unrc.edu.ar (A.F.); p.cavallo@ing.unrc.edu.ar (P.C.); 2 Instituto de Investigaciones en Tecnologías Energéticas y Materiales Avanzados, IITEMA, (CONICET-UNRC) Departemento de Biología Molecular, Facultad de Ciencias Exactas, Físico Químicas y Naturales, Universidad Nacional de Río Cuarto, Río Cuarto X5800, Argentina

**Keywords:** urea, biopolymer, controlled-release fertilizer, sulfur-crosslinked biopolymer, inverse vulcanization, nitrogen use efficiency, maize growth, sustainable agriculture

## Abstract

Modern agriculture faces major challenges due to rapid population growth, climate change, and environmental constraints. Advanced polymeric systems for controlled-release fertilizers (CRFs) are essential to address these challenges. Urea is one of the most widely used nitrogen fertilizers; however, its agronomic efficiency is limited by volatilization and losses. In this study, we report a sustainable strategy to encapsulate urea using a matrix derived from industrial sulfur waste and vegetable oil, improving agronomic efficiency while valorizing industrial residues and renewable resources. Through inverse vulcanization, a sponge-like polymer (Bp-SF) was synthesized. Two urea-loaded bio-composites (Bp-SF25U and Bp-SF32U) were also prepared. FT-IR analysis confirmed urea encapsulation and the formation of polymeric structures from sunflower oil. SEM revealed a porous morphology, while contact angle measurements confirmed the hydrophobic nature of the polymer matrix. Release kinetics showed sustained nitrogen release for more than 77 days, reaching approximately 60% cumulative release, governed by diffusion, with a fraction of urea retained within the matrix, potentially enabling prolonged nutrient availability. Pot experiments with maize showed that a lower dose of encapsulated urea (79 mg) produced similar plant growth responses to a higher dose of free urea (92 mg), indicating improved nitrogen use efficiency. These sulfur cross-linked biopolymers represent a promising strategy to enhance urea efficiency while supporting greener fertilization strategies aligned with circular economy principles.

## 1. Introduction

According to United Nations reports, the global population is increasing at an accelerated rate and is projected to peak of 10.3 billion in the mid-2080s, followed by a gradual decline to 10.2 billion by 2100 [1]. This growth places increasing pressure on agricultural systems to enhance food production while minimizing environmental impacts.

Simultaneously, the availability of arable land is declining due to urbanization, soil degradation, and climate change, making the efficient use of agricultural inputs, particularly fertilizers, increasingly critical.

Nitrogen, mainly applied as urea, is one of the most essential macronutrients for crop production and is required in large quantities to sustain high yields [2]. However, urea undergoes rapid urease-mediated hydrolysis, producing ammonia (NH_3_) and resulting in substantial volatilization losses. The residual nitrogen is subsequently oxidized by nitrifying microorganisms to nitrate (NO_3_^−^), a highly mobile form prone to leaching, while denitrification processes convert part of this nitrate into gaseous nitrogen species. Collectively, these transformations markedly decrease nitrogen use efficiency (NUE) and contribute to environmental pollution [3,4].

Maize (*Zea mays* L.) is one of the three major staple crops globally and a key source of food, feed, and industrial raw materials. Improving NUE in maize production is therefore essential to meet increasing food demand in a sustainable manner [5,6]. While conventional chemical fertilizers have historically increased maize yields, their inefficient use, high application rates, and associated environmental impacts have raised concerns regarding soil degradation and nutrient losses. Consequently, current agricultural strategies emphasize the development of environmentally friendly fertilization technologies that maintain or enhance crop productivity while reducing negative environmental impacts [7,8].

Among these strategies, controlled-release fertilizers (CRFs), also referred to as enhanced efficiency fertilizers (EEFs), have emerged as a promising approach to synchronize nutrient release with plant uptake. These systems rely on physical or chemical barriers that regulate nutrient diffusion into the soil, thereby reducing volatilization and leaching losses and improving NUE [9,10]. Polymer-coated fertilizers represent one of the most widely studied CRF technologies, in which nutrients are encapsulated within polymeric matrices acting as semi-permeable membranes [11,12]. A wide range of polymeric materials has been explored as coating or encapsulation matrices, including polyacrylamides, polyurethanes, polyesters, and cellulose-based derivatives [13,14,15,16]. However, many of these systems rely on polymers derived from non-renewable petrochemical monomers that may persist in the environment, raising concerns regarding long-term soil accumulation and sustainability. As a result, the biodegradability, environmental footprint, and raw material origin of polymer coatings remain critical challenges to be addressed [17,18]. This raises concerns regarding their environmental footprint, and alignment with sustainable and circular agricultural practices. In response to these limitations, increasing attention has been directed toward the development of biopolymeric matrices derived from renewable resources or industrial by-products, in line with circular economy principles [19]. These materials may reduce dependence on fossil resources while maintaining the functional properties required for controlled nutrient release. In this context, Cavallo et al., recently highlighted the potential of a biobased polymer derived from epoxidized soybean oil (ESO) cross-linked with citric acid as a sustainable matrix for controlled urea release [20].

Similarly, sulfur-crosslinked polymers obtained through inverse vulcanization have emerged as versatile functional materials with tunable structural and barrier properties. Previous studies have demonstrated that sulfur–vegetable oil polymers exhibit high porosity and affinity for hydrophobic compounds, enabling applications ranging from environmental remediation to controlled drug delivery [21,22,23]. More recently, their combination of hydrophobicity, porosity, and diffusion-controlled transport has motivated their exploration as encapsulation matrices in controlled-release systems, including agricultural applications [24].

Based on these considerations, this work presents the synthesis of sulfur-based biopolymers derived from sunflower oil via inverse vulcanization for use as urea encapsulation matrices. The resulting bio-composites were characterized and evaluated in terms of release kinetics and agronomic performance in maize. It is hypothesized that the sulfur-crosslinked matrix acts as a diffusion barrier that regulates water penetration and urea release due to its hydrophobic and porous structure. This controlled-release behavior is expected to reduce nitrogen losses and improve NUE compared to free urea, particularly at reduced fertilizer doses.

## 2. Results and Discussion

### 2.1. Synthesis and Characterization of Bio-Composites and Polymer

**Figure 1:**
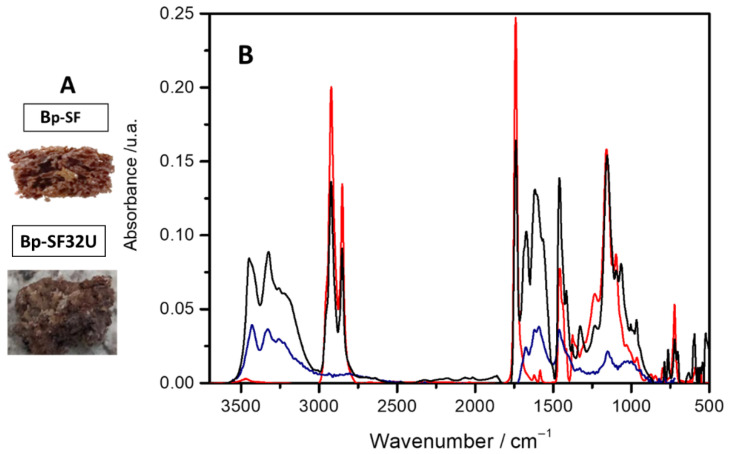
(**A**) Photographs of biopolymer and bio-composite materials; and (**B**) FT-IR of Bp-SF (red line), urea (blue line) and Bp-SF32U (black line).

The synthesis of the bio-composites Bp-SF25U and Bp-SF32U was successfully carried out through the inverse vulcanization process. By inverse vulcanization it was possible to synthesize a biopolymer (Bp-SF) based on sulfur and sunflower oil, obtaining a light brown, macroporous material with an appearance similar to that of a sponge (see Figure 1A, Bp-SF). Similar results have been reported by Farioli et al. [21,22]. In contrast, the resulting urea-loaded bio-composites exhibited distinct white inclusions, as shown in Figure 1A, Bp-SF32U. The spectra of Bp-SF, pure urea, and the Bp-SF32U bio-composite are shown in Figure 1B, and the assignment of the characteristic bands is summarized in Table 1.

As observed in the spectrum and summarized in Table 1, Bp-SF exhibits the same characteristic absorption bands previously assigned by Farioli and Worthington [21,27]. These results confirm that the expected functional groups are present, thereby indicating that the biopolymer was successfully synthesized. The FT-IR spectrum of Bp-SF32U exhibits the same characteristic bands as Bp-SF along with representative urea bands: N–H stretching at 3455 cm^−1^, C-N deformation bands at 1625 cm^−1^, and C-N stretching band at 1457 cm^−1^, which are absent in the Bp-SF spectrum. This conclusively confirms the presence of encapsulated urea, consistent with the initial visual observations.

The thermal behavior and phase transitions of the biopolymer matrix (Bp-SF) and the urea-loaded bio-composite (Bp-SF32U) were analyzed (see Figure S1). The thermogram of Bp-SF exhibits characteristic endothermic peaks in the 100–130 °C range, in agreement with previous reports on sulfur–vegetable oil copolymers [21,30,31]. These signals are attributed to the melting of different monoclinic sulfur domains and sulfur species strongly interacting within the polymer network, where confinement effects can lead to a depression of the melting temperature compared to bulk elemental sulfur [21]. Both Bp-SF and Bp-SF32U display the characteristic sulfur melting peak at ~119 °C with comparable intensity, indicating a similar content of free sulfur in both materials [32]. Consistently, the integration of the DSC endothermic peaks in the 100–150 °C range yields a fraction of free sulfur in good agreement with previously reported values for sunflower oil biopolymers of approximately 33% [21]. In the case of Bp-SF32U, an additional broad endothermic signal centered at ~135 °C is observed, which is attributed to the melting of urea dispersed within the hydrophobic micro- and nanoporous polymer matrix [20]. The broadening of this transition, compared to pure crystalline urea, suggests a homogeneous physical distribution of the nutrient within the cross-linked network. Furthermore, thermal events observed above ~160 °C are assigned to the onset of urea thermal decomposition [33]. This assignment is consistent with literature reports indicating that urea melts at ~133–135 °C and undergoes progressive thermal decomposition at temperatures above 152–160 °C, with an onset typically observed near 175 °C, leading to the formation of species such as biuret and cyanuric derivatives [33,34]. Overall, these results confirm the successful incorporation of urea into the biopolymeric matrix and demonstrate that the resulting bio-composites remain thermally stable up to 175 °C [20].

SEM imaging and EDX analysis were carried out to evaluate the morphology and confirm the presence of urea in the bio-composites (see Figure S2). These images allowed the identification, by visual inspection, of domains associated with urea crystals in the bio-composites. SEM micrographs reveal that all materials exhibit an inherently irregular and heterogeneous morphology, which is consistent with the nature of materials obtained via inverse vulcanization [22]. However, a clear morphological distinction can be identified when comparing the base biopolymer (Bp-SF) with the bio-composites (Bp-SF25U and Bp-SF32U), Bp-SF displays a more open and porous structure, whereas the bio-composites exhibit a more compact morphology. This feature can be attributed to the incorporation of urea into the matrix, as its presence likely occupies, partially or completely, the void spaces (pores) within the biopolymeric structure, resulting in a denser material. Furthermore, in the highest urea content biocomposite (Bp-SF32U), crystalline domains can be clearly observed on the material surface, providing further evidence of successful urea incorporation, as supported by EDX analysis. The qualitative observations obtained from SEM provide consistent and meaningful insights into the morphological differences among the materials, highlighting the structural effect of urea incorporation within the biopolymer.

Mass balance analysis showed minimal mass losses during synthesis: 1.61% for Bp-SF25U, and 1.41% for Bp-SF32U, indicating that no significant loss of mass occurs under the applied conditions. Consistently, DSC thermograms of the bio-composites display the characteristic melting transition of urea at 135 °C without clear evidence of extensive thermal decomposition at 160 °C, while FT-IR spectra retain the main vibrational bands associated with urea. In addition, EDX analysis confirms the presence of nitrogen in the bio-composites. Altogether, these results provide consistent and conclusive evidence of the successful synthesis of the materials and the effective incorporation and retention of urea within the polymer matrix.

### 2.2. Urea Release Kinetics from Bio-Composites

The contact angle measurement reveals that Bp-SF exhibits a significant hydrophobic behavior, with a contact angle of 121.3 ± 1.3°, which is attributed to the nonpolar nature of the sulfur–oil network, consistently with previous reports [21,22]. The incorporation of urea reduced the contact angle to 110.5 ± 1.7° (Bp-SF25U) and 101.7 ± 1.5° (Bp-SF32U), indicating increased surface polarity and improved wettability. This enhanced wettability facilitates water penetration into the polymer matrix, promoting urea dissolution and diffusion. The decrease in contact angle suggests improved surface wettability, which may favor water uptake at the matrix interface and contribute to the diffusion-controlled release of urea.

**Figure 2:**
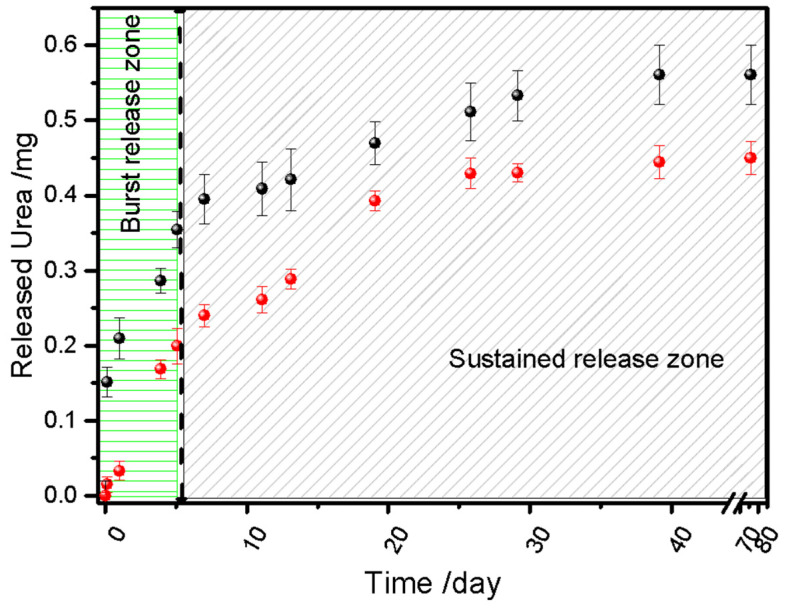
Mass of Urea released vs. time: Bp-SF32U (black) and Bp-SF25U (red).

The urea release from the bio-composites Bp-SF25U and Bp-SF32U evaluated in water over 77 days, are presented in Figure 2. Both systems exhibited an initial burst release during the first 6 days (burst release zone), followed by a second stage, with a markedly slower release rate (sustained release zone) suggesting a two-stage release mechanism. This behavior is characteristic of slow-release systems, where the biopolymeric matrix regulates nutrient availability. The initial burst release could be ascribed to Fickian diffusion of urea located at or near the surface of the biopolymeric matrix, where diffusional path lengths are minimal and the resistance to mass transfer is low. As the system evolves, the release profile transitions into a sustained regime, primarily governed by the diffusion of urea entrapped within the inner domains of the bio-composites. In this stage, transport occurs through a thicker and more tortuous polymeric barrier, imposing higher diffusional resistance and effectively retarding the outward flux. Comparable biphasic release mechanisms have been described by Cavallo et al., using hydrophobic ESO–citric polymer matrix to encapsulate urea. This composite [20] exhibited a faster initial burst release (≈14–16% within the first 2 h) attributed to the rapid dissolution of surface-localized urea crystallites, as confirmed by SEM analysis. This stage was followed by a sustained release phase governed by diffusion through an interconnected network of micro- and nanopores formed during urea migration, ensuring prolonged nitrogen delivery. This behavior has been observed in other polymer-based delivery systems [20] such as poly(lactic-co-glycolic acid) microparticles loaded with diprophylline, where the burst phase was attributed to the rapid dissolution of drug crystals directly accessible at the particle surface. The modulation of release kinetics is therefore intrinsically linked to the microstructural organization of the polymeric matrix, the distribution of the active agent within the network, and the evolving concentration gradient driving mass transport. The results suggest that the release of encapsulated urea occurs gradually and slowly in both cases. Furthermore, the results indicate that urea is available during the 69 days of the vegetative growth cycle of maize, implying that a single fertilizer application at sowing could be sufficient. This approach could reduce operational costs and labor requirements by eliminating the need for multiple fertilizer applications during crop development.

**Figure 3:**
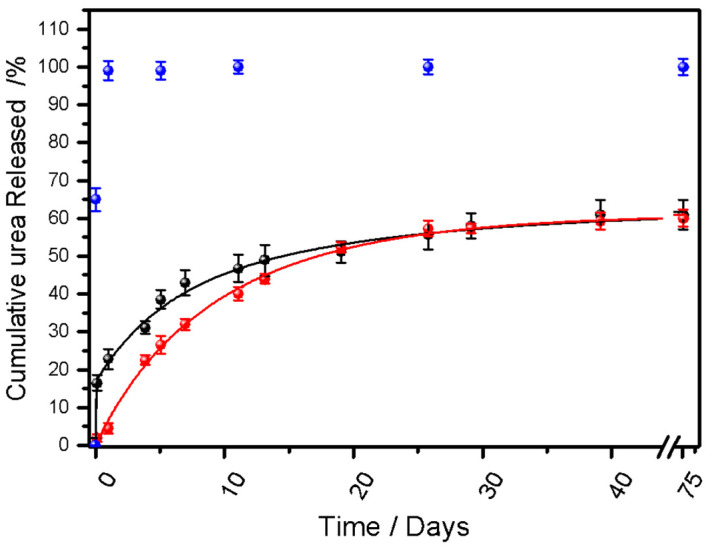
Profiles of Cumulative percentage urea release of: non-encapsulated (blue circles) and encapsulated urea samples (Bp-SF32U (black circles) and Bp-SF25U (red circles) over time. Error bars indicate the standard deviation of three independent measurements.

Figure 3 depicts the percentage of cumulative urea release profiles over the 77-day evaluation period for Bp-SF32U and Bp-SF25U. It can be observed neither of the bio-composites achieved complete release of the encapsulated urea within this timeframe, instead both bio-composites released up to 60% of the loaded urea. In contrast, free urea exhibited complete release within less than 1 day. Nevertheless, the cumulative release values correlated well with the initial urea loading of each formulation, confirming both the efficiency of the encapsulation process and the stability of the biopolymeric matrices. During the first 6 days, Bp-SF32U exhibited a higher release rate (6.5%/day) compared to Bp-SF25U, probably due to the release of urea located at the surface of the bio-composite. In contrast, Bp-SF25U displayed an almost constant release rate (4.2%/day) up to around day 6. By the end of the experimental period, both systems presented an exponential profile. Similar results have been found in cellulose-based controlled-release systems, which release 45.11% of the encapsulated urea within 5 days [35]. Moreover, Poly(acrylic acid-co-acrylamide) grafted on starch releases 70% of N after 21 days [36]. These findings highlight that a higher urea loading does not necessarily translate into greater release within the same time window, underscoring the critical influence of the polymeric matrix structure and diffusional resistance in governing the release kinetics.

The incomplete release observed at the end of the study may be due to urea confined in the inner regions of the biopolymeric matrix, limited accessibility, or a decreased concentration gradient reducing the diffusion rate. Far from being a disadvantage, this behavior can be beneficial in prolonged agricultural applications, maintaining a reserve of fertilizer for later crop growth stages and supporting more sustained and efficient nutrition. Furthermore, the release profile analysis indicates that urea remains available throughout the 69-day vegetative growth cycle of maize (*Zea mays* L.) [37,38], suggesting that a single application at the time of sowing could be sufficient to meet the crop’s nitrogen demand. This strategy would eliminate the need for multiple fertilization events during different phenological stages, thereby reducing operational costs and labor requirements associated with macronutrient application.

**Figure 4:**
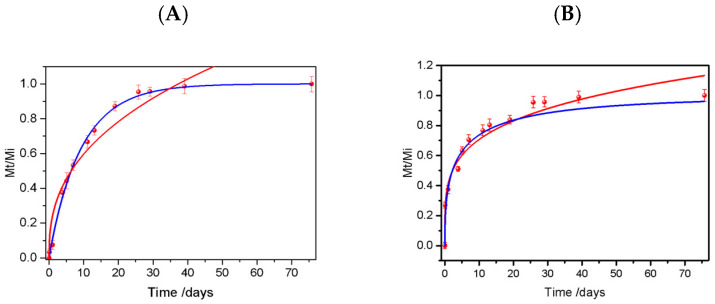
Kinetic of the urea release profiles for: (**A**) Bp-SF25U and (**B**) Bp-SF32U fitting employing: Korsmeyer–Peppas (red line) and Weibull (blue line).

Overall, the results demonstrate that bio-composites function as efficient matrices for controlled urea release, with performance tunable through material formulation and polymer-to-fertilizer ratio. Consequently, this approach has the potential to decrease fertilization costs, minimize labor inputs, and mitigate nutrient losses caused by leaching and volatilization, contributing to more sustainable agricultural practices. The kinetic studies of the urea release can be analyzed using the Korsmeyer–Peppas and Weibull models. The data of the Bp-SF32U and Bp-SF25U were fitted using both models in Figure 4A,B, respectively. Also, the parameters of the mathematical models for urea release of the Bp-SF25U and Bp-SF32U are shown in Table 2.

In Bp-SF25U, the Korsmeyer–Peppas model yielded a diffusion exponent n = 0.39, characteristic of a quasi-Fickian diffusion mechanism [39]. This behavior suggests that urea molecules navigate a tortuous path within the hydrophobic matrix, where the sulfur-oil network ensures that the diffusion rate is limited by water penetration into the internal pores [40]. In contrast, Bp-SF32U exhibited n = 0.23, indicating a shift in diffusion towards a parabolic release profile [41], and suggesting that the biopolymer matrix with a higher urea content possesses larger interconnected channels, which facilitate a burst release effect. This initial rapid stage is driven by the immediate dissolution of surface-localized urea that is easily accessible to the aqueous medium. Moreover, the Weibull model further supports this interpretation. The β = 0.44 for Bp-SF32U suggests a faster initial release followed by stabilization in contrast to the β value obtained for Bp-SF25U (0.97) [42]. This significant decrease in β for Bp-SF32U indicates that once the surface urea is depleted, the release rate decreases as the process becomes limited by the diffusion of urea located deep within the internal core of the polymeric matrix. In this second stage, the dissolved nutrient must navigate a much longer and more resistant path through the hydrophobic structure to reach the surface [43]. The results obtained are consistent with previous studies demonstrating that the release control in polymeric matrices is strongly influenced by the matrix structure and the quantity of the active compound loaded. In slow-release hydrogel systems, release times can extend for days, as reported in recent studies where the hydrogel’s crosslinked structure and water retention capacity determine the release kinetics, as reported by Mandal et al. [44]. In studies using starch-alginate matrices [45], urea release follows a Fickian diffusion model, with n < 0.43, similar to that observed for Bp-SF32U. However, the matrix’s water retention capacity and the presence of diffusion channels play a fundamental role in these systems, which could explain the differences in release rates. Similarly, urea-coated films show that the release time is determined by the film thickness and permeability [46]. In this context, the polymer matrix of epoxidized soybean oil exhibited a stable, diffusion-controlled urea release governed by the hydrophobic and low-swelling nature of the biobased polymer matrix. The high hydrophobicity of the sulfur-oil matrix (contact angle 121.3°) provides superior mass-transfer resistance compared to traditional hydrophilic systems, ensuring that the driving force for urea release remains controlled over the 77-day evaluation [47]. Finally, in systems using mineral coatings, such as urea coated with wax and zeolite [48], the Weibull model accurately describes diffusion-controlled release, which is consistent with the results observed in this study.

### 2.3. Soil Properties Analysis

The soil used for the maize growth experiment was analyzed to determine its physical parameters, as presented in Table 3.

The soil used in the maize growth experiment is characterized by low electrical conductivity (147.8 µS cm^−1^) and a slightly acidic pH of 5.90, indicating a non-saline, agriculturally suitable substrate with no significant salinity stress for crops like maize. This low EC value falls well below thresholds associated with salinity problems (where >0.4 dS m^−1^ begins to indicate mild salinity) and are typical of productive agricultural soils with minimal soluble salt accumulation. A pH of 5.9 is within the slightly acidic range commonly observed in cultivated soils and is generally acceptable for nutrient availability in many crops [41]. Similar studies of agricultural fields have reported slightly acidic pH and low EC values when assessing soil conditions for plant growth, supporting the finding that these measured properties are within typical ranges for non-saline, productive soils [42]. Overall, these values allow for a comparison with other soil profiles used in crop studies and indicate that the soil environment was appropriate for evaluating maize response under controlled fertilization treatments.

### 2.4. Germination of Maize Seedlings

**Figure 5:**
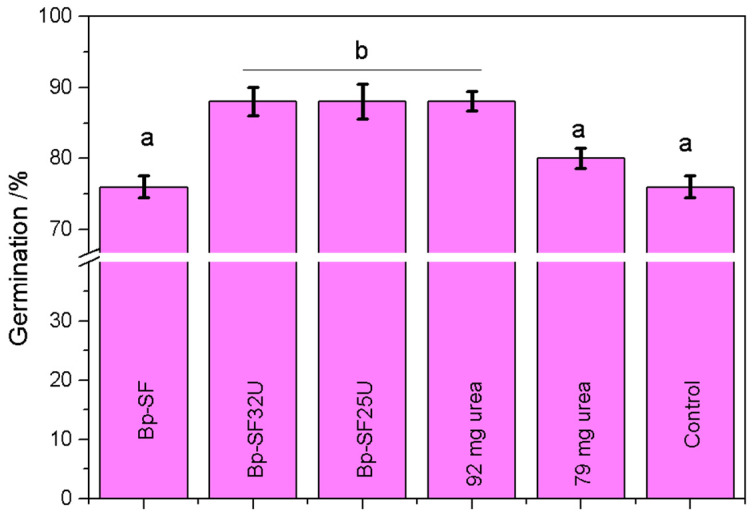
Germination percentage of each treatment. Each value represents the mean of three independent replicates, each consisting of five pots per treatment with five seeds per pot. Mean ± SE followed by different letter is statistically significant at *p* < 0.05 by ANOVA post Tukey test.

The germination promotion effect of the Bp-SF25U and Bp-SF32U fertilizer on maize seedling was evaluated under laboratory conditions, and the results are presented in Figure 5. As can be seen in Figure 5, none of the applied treatments reached a 100% germination percentage (% Germ), but the highest % Germ (88%) was achieved by treatments Bp-SF32U (containing 92 mg of urea), Bp-SF25U (containing 79 mg of urea) and 92 mg of free urea. The control treatment reached 76% of germination, a value identical to that observed using Bp-SF biopolymer alone. Importantly, none of the urea doses tested, whether free or encapsulated, negatively affected seed germination, confirming the safety of these formulations during early maize development. The biopolymer without urea showed no negative effect on germination. Moreover, the encapsulated urea formulations Bp-SF25U and Bp-SF32U enhanced maize seed germination compared to the control and the biopolymer alone. Notably, Bp-SF25U, with a lower urea load (79 mg), achieved the same % Germ (88%) as free urea applied at a higher dose (92 mg), indicating that urea encapsulation preserves nitrogen effectiveness while reducing the required fertilizer amount. Similarly, Bp-SF32U produced comparable germination results, confirming that lower or equivalent nitrogen doses can generate the same biological response when delivered through the biopolymer matrix.

### 2.5. Effect of Biopolymer on Plant Growth in Pot Condition

**Figure 6:**
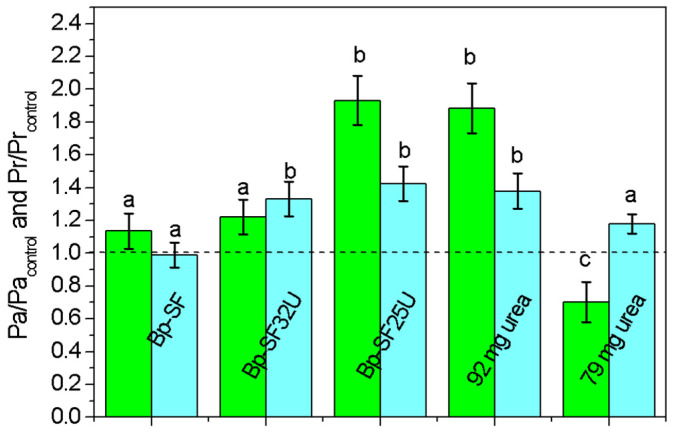
Dry matter Pa (light blue) and dry matter Pr (green) of the applied treatments compared to the control treatment. The dashed line corresponds to the value of the control treatment. Mean ± SD followed by different letter is statistically significant at *p* < 0.05 by ANOVA post Tukey test.

The effect of the different treatments on the maize growth was evaluated by measuring the biomass response of both aerial (Pa) and root (Pr) biomass in comparison with the control treatment. Figure 6 shows that the Bp-SF32U and Bp-SF25U treatments produced a more pronounced positive effect, particularly on Pr, which reached values approximately 1.23 and 1.93-fold higher than the control, respectively. In both treatments, Pa also increased, reaching approximately 1.33 and 1.42 fold higher than the control, respectively. The treatments containing free urea (79 mg and 92 mg of urea) exhibited contrasting responses. Under 92 mg of free urea, Pr increased to approximately 1.88-fold, whereas Pa showed a moderate increase of approximately 1.37. Conversely, in the treatment using 79 mg of free urea, the Pr biomass dropped below unity (≈0.7), while Pa remained slightly above the control (≈1.17).

Regarding the average Pr statistics, all treatments involving the encapsulation of urea in the Bp-SF matrix presented appreciable differences both among themselves and compared to the control. Overall, the results indicate that the Bp-SF25U treatment promotes plant growth more effectively than Bp-SF alone or free urea. The marked increase in aerial biomass under Bp-SF25U and 92 mg of free urea suggests enhanced nutrient availability or improved nutrient uptake efficiency when Bp-SF is combined with urea. This supports the assumption that the biopolymer matrix does not have a negative effect on the growth of the maize root system; on the contrary, it appears to favor it. This observation is consistent with the role of Sulfur (S), as an essential mineral nutrient for plants, required for the synthesis of proteins (Met and Cys), oligopeptides (glutathione, phytochelatin), chlorophyll, enzymes, vitamins (thiamine, biotin) and various secondary metabolites (such as glucosinolates.), impacting overall metabolic and photosynthetic processes [43,44]. The consistent increase observed in Pa across these treatments further supports a stimulatory effect on root development, which may contribute to improved nutrient absorption and overall plant vigor.

The reduced Pr biomass observed in the 79 mg of free urea treatment indicates that this urea concentration alone may leach or volatilize when not encapsulated within by the polymer matrix. In contrast, when urea is incorporated into the bio-composites (Bp-SF32U and Bp-SF25U), this negative effect is mitigated, suggesting that the polymer regulates nutrient release and reduces the nitrogen losses. The greater effect on Pr observed for Bp-SF25U could be due to the fact that controlled-release or polymer-coated urea often improves both root morphology and above-ground growth by synchronizing nitrogen availability with plant demand. The polymer matrices can reduce the initial spike of available urea and prolong N release, avoiding high local N concentrations that can cause stress. This buffering effect is consistent with our finding that the 79 mg of free urea treatment reduced the aerial biomass (Pa ≈ 0.7) whereas the same nominal urea dose embedded in Bp-SF (Bp-SF25U) did not cause such a reduction and instead increased both Pr and Pa. Similar behavior has been demonstrated in studies showing that polymer-coated urea (PCU) increases root growth and nitrogen use efficiency in the super-large-panicle indica/japonica hybrid rice cultivar Yongyou 1540, thereby promoting greater shoot biomass and yield [45].

The variability observed in some treatments may reflect differential plant responses to nutrient availability or heterogeneity in nutrient release dynamics. Nevertheless, the overall pattern shows that polymer–urea combinations promote both aerial and root growth beyond the levels achieved by the individual components.

Several agronomic and physiological studies have also reported a clear benefit of slow-release formulations on root biomass and activity: a steadier N supply promotes root proliferation and enzymatic activity, improving soil exploration and nutrient acquisition. This is consistent with our observation of increased Pr across polymer-containing treatments and suggests that the polymer matrix not only prevents toxicity but actively promotes root development, which in turn supports the observed gains in shoot biomass.

Despite the promising agronomic performance of the Bp-SF bio-composites, this study presents certain limitations that should be acknowledged. First, nutrient release experiments were conducted under simplified aqueous conditions, which primarily evaluate the material’s diffusion-controlled mechanism but do not fully represent the complex interactions of soil environments, such as microbial activity and varying moisture levels. Additionally, while urea incorporation was confirmed via FT-IR and EDX, the total content was estimated indirectly through mass balance, and specific nitrogen dynamics—including direct quantification of leaching and volatilization—remain to be characterized in soil-based systems. A notable observation was that approximately 40% of the encapsulated urea remained unreleased after the 77-day evaluation period. While this could potentially serve as a nutrient reserve for later phenological stages or perennial crops, its long-term bioavailability and the environmental fate of the remaining matrix require further investigation. Furthermore, although the plant growth assays demonstrated significant biomass enhancement, extrapolation to field-scale performance requires caution due to the controlled nature of the greenhouse conditions. Future research will prioritize soil-based release studies, direct nitrogen loss measurements, long-term degradation rates and extended field trials across diverse climates and soil types to fully establish the scalability of these bio-based fertilizers. Nonetheless, the available evidence supports the environmental viability of these materials as sustainable alternatives to conventional petrochemical polymers.

Overall, the results demonstrate the successful development of sulfur-based bio-composites with suitable structural and thermal properties for their intended application. While the direct quantification of degradation rates was not the primary focus of this specific study, the environmental breakdown and safety of these biopolymeric matrices are supported by established chemical principles and our recent toxicological assessments [49]. Moreover, in soil environments, sulfur species are biologically oxidized to sulfate by sulfur-oxidizing microorganisms as part of the biogeochemical sulfur cycle [50]. Previous literature confirms that sulfur–vegetable oil copolymers exhibit significant biodegradation potential, studies have demonstrated that soil microorganisms can oxidize the sulfur within the matrix into sulfate, an essential plant nutrient, at rates significantly higher than those of elemental sulfur alone [3,50,51]. As established by Valle et al., the carbon backbone derived from vegetable oils in these biopolymers acts as an energy source for heterotrophic soil microorganisms, such as Aspergillus niger, which facilitates microbial growth and enhances the oxidation process. Furthermore, inverse vulcanized polymers contain dynamic S–S bonds that undergo cleavage and rearrangement, enabling progressive depolymerization into lower-molecular-weight species [52]. Since triglyceride-derived moieties are used as comonomers, the resulting organic compounds are inherently susceptible to microbial degradation [53]. Bio-compatibility assays using the *Allium cepa* test confirmed that these sulfur biopolymers and their composites are non-cytotoxic and non-genotoxic to eukaryotic cells, demonstrating that no harmful residues are released [49].

## 3. Materials and Methods

### 3.1. Materials

Biopolymers based on sunflower oil (SF) and elemental sulfur (S) were synthesized via inverse vulcanization. Commercial sunflower oil (Natura^®^, AGD, General Deheza, Cordoba, Argentina), and elemental sulfur (Sigma-Aldrich, Buenos Aires, Argentina, purity > 99%) were used as comonomers. A 50:50 S:SF mass ratio was employed to produce the cross-linked biopolymer (Bp-SF). Granular urea was purchased from Cicarelli, (San Lorenzo, Argentina).

### 3.2. Methods

#### 3.2.1. Synthesis and Characterization Urea Loaded Bio-Composites: Bp-SF-Urea

The bio-composites were synthesized according to the procedure reported by Farioli et al. [21]; with specific modifications to ensure efficient nutrient encapsulation. The reaction was conducted in a 250 mL glass reactor open to the atmosphere and equipped with a mechanical stirrer to ensure uniform heat distribution and phase mixing. Initially, sunflower oil and urea were mixed and heated to 160 °C under continuous stirring for approximately 7 min. This mixture was then added to the reactor containing molten sulfur (previously stabilized at 160 °C), maintaining a constant stirring rate of 900 rpm. The reaction was performed under an air atmosphere, without the use of inert gases. The progress of the polymerization was evidenced by a gradual and significant increase in the viscosity and volume of the reaction system, which became clearly noticeable approximately 20 min after the addition of the oil–urea phase. After that, stirring was stopped, the reactor was removed from the heating source, and the resulting bio-composites were allowed to cool to room temperature under static conditions. Two bio-composites were synthesized, varying the mass of urea and keeping the mass ratio of sunflower oil to sulfur constant at 50:50, these were designated as Bp-SF25U (0.25 g Urea/g Bp-SF) and Bp-SF32U (0.32 g urea/g Bp-SF).

Urea content was indirectly estimated by a mass balance approach. The total mass of the reaction system was recorded before and after synthesis, and the mass loss during the process was calculated.

The percentage of urea contained in each of the biopolymers used in the tests was calculated using Equation (1). The mass of each reactant employed in the reaction is shown in Table 4.

(1) $$U=\frac{mb\cdot mu}{mt}$$

where mb is the mass of the biopolymer used in the growth assay, mu is the mass of urea encapsulated during synthesis, and the total mass (mt) is the sum of the masses of the reactants used to obtain the bio-composite (mass of S, mass of vegetable oil, and mass of urea).

#### 3.2.2. FTIR-ATR Spectroscopy

Successful urea encapsulation within the Bp-SF matrix was confirmed by Fourier Transform Infrared (FTIR-ATR) spectroscopy. The FTIR-ATR spectroscopy experiment was carried out in absorbance mode using a Bruker Tensor 27 spectrometer (Bruker, Ettlingen, Germany). The spectra were obtained with 30 scans per spectrum at a spectral resolution of 4 cm^−1^ in the wavenumber range from 4000 to 500 cm^−1^. As a reference, the background spectrum of air was collected before the acquisition of each sample spectrum.

#### 3.2.3. Differential Scanning Calorimetry

The thermoresponsive behavior of the materials was evaluated by differential scanning calorimetry (DSC) using a Netzsch DSC 204 F1 Phoenix calorimeter (Netzsch, Selb, Germany) equipped with a cooling system. Samples (c.a. 3.5 mg) sealed in aluminum pans were subjected to heating from 80 to 175 °C at a heating rate of 5 °C/min under a nitrogen atmosphere (50 mL/min). This protocol was employed to assess the influence of material composition on the thermal properties.

#### 3.2.4. Contact Angle Measurement

Surface wettability was evaluated through static water contact angle measurements using a Theta Flow optical tensiometer (Biolin Scientific, Gothenburg, Sweden). Due to the inherently macroporous and sponge-like nature of the sulfur–vegetable oil biopolymers, samples were prepared as irregular cubic fragments (≈1 cm × 1 cm × 1 cm) cut from the bulk material using a precision scalpel, aiming to obtain surfaces as regular and planar as possible. Surface quality and alignment were verified via the tensiometer’s optical system prior to liquid deposition to mitigate the influence of surface irregularities. Droplets of bi-distilled water (5 μL) were deposited on the surface at room temperature. The resulting contact angles were recorded and analyzed using OneAttension^®^ software (version 4.0, Biolin Scientific), with each reported value representing the average of five photographs. To ensure reproducibility and account for the structural heterogeneity of the bio-composites, the informed contact measurements were performed in triplicate, and for each measurement, the reported value was the average of five photographs analyzed.

#### 3.2.5. Scanning Electron Microscopy (SEM) and Energy-Dispersive X-Ray Analysis (EDX)

The morphology of the materials was analyzed by scanning electron microscopy (SEM) using a Carl Zeiss EVO MA10 microscope (Carl Zeiss, Oberkochen, Germany) equipped with an energy-dispersive X-ray (EDX) detector. Prior to observation, the samples were sputter-coated with a thin gold layer for 90 s at 40 mA under an argon atmosphere (0.4 mbar). SEM images were acquired at an accelerating voltage of 3 kV. In addition, EDX analysis (at 3 kV, acquisition time of 60 s) was performed to determine the elemental composition of the samples, confirming the presence of sulfur (S), and nitrogen (N).

#### 3.2.6. Controlled Release Analysis

In order to study the release behavior of the encapsulated urea, samples were immersed in aqueous solutions. For the release analysis, 3 g of each bio-composite sample were immersed in 150 mL of distilled water in a sealed vessel. The samples were maintained at a constant temperature of 25 °C using a thermostatic bath. The ratio of bio-composite to water was carefully selected to ensure sink conditions throughout the 77-day evaluation. Considering the high solubility of urea in water (approx. 1080 g/L at 20 °C), the maximum theoretical concentration in our system (~6.67 g/L) remains below 0.7% of the saturation limit. This ensures that the concentration gradient remains the primary driving force for release, preventing saturation effects that could artificially alter the diffusion profile [47,54]. Furthermore, this volume was optimized to maintain urea concentrations within the ideal linear calibration range (0.1–1.0 absorbance units) of the p-dimethylaminobenzaldehyde (DMAB) spectrophotometric method without requiring excessive dilutions [55]. At predetermined time intervals, 1 mL aliquots were collected, and the urea concentration was quantified by UV-visible spectroscopy using a Varian Cary^®^ 50 UV-Vis spectrophotometer (Agilent Technologies, Santa Clara, CA, USA). Urea quantification was performed via derivatization: each 1 mL aliquot was reacted with 1.8 mL of a solution containing 0.3 g of p-dimethylaminobenzaldehyde (DMAB), 100 mL of acetonitrile, and 3.6 mL of concentrated hydrochloric acid (37% *w*/*w*) [20]. This reaction produced a yellow-colored solution, the absorbance of which was measured at 420 nm.

The urea release mechanisms from the Bp-SF bio-composites were quantitatively analyzed by fitting the experimental release data to two well-established kinetic models: the Korsmeyer–Peppas [46,47] model. The cumulative release data were fitted to the Korsmeyer–Peppas equation (Equation (2)):

(2) $$\frac{M_{t}}{M_{i}}=k\cdot t^{n}$$

where Mt/Mi is the fraction of urea released at time t, k is the kinetic constant, and n is the diffusion exponent indicative of the release mechanism. The value of n was interpreted according to established criteria for spherical matrices.

Additionally, the release data were fitted using the Weibull model [48] to the Weibull Equation (3):

(3) $$\frac{M_{t}}{M_{i}}=1-e^{-{\left(\frac{t}{\tau }\right)}^{\beta }}$$

where τ is a scale parameter representing the process time, and β is a shape parameter that characterizes the release curve as exponential (β = 1), parabolic (β < 1), or sigmoidal (β > 1). Model fitting and parameter estimation were performed using nonlinear least-squares regression in OriginPro^®^ (version 9.1).

#### 3.2.7. Soil Properties

The soil used for maize growth experiments was previously analyzed to determine its physical and chemical parameters such as conductivity, pH, soil color and odor.

#### 3.2.8. Pot Experiment

The experiment aimed to evaluate the impact of the biopolymeric matrix Bp-SF and the two bio-composites, Bp-SF25U and Bp-SF32U, on the plant growth. Growth experiments were conducted using *Zea mays* plants, for this, 1 L pots (15 cm of diameter) containing 1 kg of soil from a field in the Río Cuarto region were used. The pots were maintained for 69 days, until the plants completed the vegetative phase.

The objective of this study was to design a controlled-release system under realistic agronomic conditions, ensuring that the biopolymer/urea ratios reflected feasible formulations for field applications. Fertilizer application rates were scaled according to the surface area of the pots and expressed in kg ha^−1^, following a common approach in pot experiments to ensure comparable nutrient input per unit area. The pot experiment was designed to provide a controlled and comparative assessment of the materials under standardized conditions and does not imply direct extrapolation to field conditions. The fertilization rate was based on field-relevant applications reported in the literature, corresponding to a conventional dose of 75 kg urea ha^−1^ [20]. Considering that the experiments were conducted in cylindrical pots with a diameter of 15 cm, the applied urea amounts resulted in equivalent surface-based doses of 45 and 52 kg urea ha^−1^ for Bp-SF25U and Bp-SF32U, respectively. These values represent reductions of approximately 40% and 30% relative to the recommended urea dose in order to evaluate the effect of the encapsulated urea.

#### 3.2.9. Preparation of Maize Seeds

The seeds were sterilized for 20 min in a 2% sodium hypochlorite solution to eliminate surface microbial contaminants and subsequently rinsed with distilled water.

#### 3.2.10. Seed Germination and Early Seedling Growth

The effect of bio-composite fertilizers on seed germination and early-stage seedling growth of maize was investigated by comparison with neat urea treatment and a urea-free control. The experiments were conducted with six treatments using three independent replicates, each consisting of five pots per treatment with five seeds per pot: Treatment 1: Bp-SF, Treatment 2: 0.316 g of Bp-SF25U (79 mg urea encapsulated Bp-SF), Treatment 3: 0.288 g Bp-SF32U (92 mg urea encapsulated Bp-SF), Treatment 4: 92 mg of free urea, Treatment 5: 79 mg of free urea, and Treatment 6: Control (without urea or biopolymer).

This approach enabled a direct comparison of plant growth performance across treatments, providing insight into the effect of the biopolymer matrix with urea encapsulation relative to free urea applications under controlled conditions. The pots were placed in growth chambers under controlled conditions of temperature (24–25 °C), ambient humidity (60–70%), photoperiod (16 h of light, 8 h of darkness) and luminescence intensity (10,000–11,000 lux). During the germination experiment, the pots were watered with distilled water two times a day, using a volume of 50 mL.

The final germination percentage was obtained by dividing the final number of germinated seeds in each pot by the total number of sown seeds, and multiplying by 100. This calculation was performed for all treatments and the control.

#### 3.2.11. Vegetative Growth Measurements

To investigate the effect of different treatments on maize growth performance in terms of aerial biomass (leaves, stems, and branches) and root biomass (roots). Six treatments were established with 10 pots per treatment, arranged in two independent experiments (5 pots per treatment): Control, two treatments of urea-free urea (79 mg and 92 mg free urea), Bp-SF25U, Bp-SF32U and Bp-SF. In each treatment, similar masses of the bio-composite/biopolymer were incorporated along with the seeds: 0.288 g of Bp-SF32U (containing 92 mg of free urea), and 0.316 g of Bp-SF25U (containing 79 mg free urea), or 0.294 g of Bp-SF. Experiments were conducted in growth chambers under controlled conditions of temperature (24–25 °C), ambient humidity (60–70%), photoperiod (16 h of light, 8 h of darkness) and light intensity (10,000–11,000 lux). After germination, during the vegetative growth experiment, the pots were watered with distilled water after germination 3 times per week with 100 mL of distilled water per irrigation.

After 69 days of growth, the plants were carefully harvested by removing them from the pots. The aerial parts and roots were carefully washed with deionized water to remove any surface soil or dust deposited. The average dry weight was obtained after drying the plants in an oven at 60 °C for 48 h until a constant weight was reached.

#### 3.2.12. Statistical Analysis

The data were analyzed using one-way analysis of variance (ANOVA) at a confidence level of α = 0.05% to determine significant differences in crop responses to the treatments. When statistical significance was found (*p* ≤ 0.05), a comparison of the means was carried out using the Tukey test (in OriginPro^®^ version 9.1).

## 4. Conclusions

Sulfur-crosslinked biopolymeric matrices synthesized via inverse vulcanization proved to be effective materials for controlled urea delivery. The Bp-SF-based bio-composites exhibited a hydrophobic and porous structure that enabled diffusion-controlled nitrogen release for over 77 days, with cumulative urea release of approximately 60% of total urea, Korsmeyer–Peppas exponents (n < 0.40) and Weibull shape parameters (β < 1), confirmed a Fickian transport mechanism.

Encapsulated formulations (Bp-SF25U and Bp-SF32U) enhanced maize germination up to 88%, compared to 76% for the control and the biopolymer alone, achieving responses comparable to free urea despite using reduced nitrogen doses. Pot experiments showed significant increases in plant biomass, with root biomass reaching up to 1.93-fold and shoot biomass up to 1.42-fold relative to the control.

Overall, the sulfur–sunflower oil biopolymeric matrix provides a stable and sustainable platform for diffusion-governed urea release. The use of industrial waste (elemental sulfur) and renewable oils as low-cost precursors, combined with the potential to reduce application frequency, offers a promising and potentially cost-effective perspective for improving agronomic performance while supporting circular economy-based fertilization strategies.

## Figures and Tables

**Table 1:** FT-IR assignment of the characteristic bands of urea, Bp-SF and Bp-SF32U.

Wavenumber (cm^−1^)	Assignation	Reference
~3455	–N-H stretching	[25]
~2954	C–H stretching	[26]
~1700	C=O stretching	[22]
~1625	C–N deformation	[25]
~1457	C–N stretching	[25]
~1300–1000	C–O stretching	[27]
930	S–S stretching	[21,28,29]
790	C–S stretching	[28,29]
~500–400	S–S stretching	[28,29]

**Table 2:** Parameters of mathematical models for urea release of the Bp-SF25U and Bp-SF32U bio-composites.

Sample	Mathematical Models	Parameters		
Korsmeyer–Peppas *		k	n	R^2^
Bp-SF25U		0.24 ± 0.040	0.39 ± 0.051	0.973
Bp-SF32U		0.41 ± 0.021	0.23± 0.017	0.975
Weibull *		α	β	R^2^
Bp-SF25U		9.3 ± 0.24	0.96 ± 0.037	0.999
Bp-SF32U		5.2 ± 0.73	0.44 ± 0.051	0.962

* k is the release constant; n is the diffusional exponent used to distinguish the mechanism of drug release; α is time scale parameter; β is shape parameter.

**Table 3:** Physical parameters of the soil used for the corn crop growth test.

Physical Parameters	
Conductivity	147.8 μs
pH	5.90
Soil color	Dark brown
Soil odor	Odorless

**Table 4:** Mass of reactive employed to obtain the biopolymer Bp-SF and the bio-composites Bp-SF25U and Bp-SF32U and the percentage of urea in the bio-composites.

Bio-Composites	Sulfur (Grams)	SF Oil (Grams)	Urea (Grams)	%U
Bp-SF	30	30	0.00	0.00%
Bp-SF25U	30	30	20.00	25.00%
Bp-SF32U	30	30	28.00	31.81%

## Data Availability

The data that support the findings of this study are available from the corresponding author upon reasonable request.

## Supplementary Materials

The following supporting information can be downloaded at: https://www.mdpi.com/article/10.3390/ijms27093863/s1.

## Abbreviations

The following abbreviations are used in this manuscript:

CRFs	controlled-release fertilizers
Bp-SF	Biopolymer obtained by inverser vulcanization of sunflower oil and S
Bp-SF25U	Bio-composite obtained using Bp-SF and 25% of urea
Bp-SF32U	Bio-composite obtained using Bp-SF and 32% of urea
ESO	Expoxidated sunflower oil
SF	sunflower oil
Pa	Aerial biomass
Pr	Root biomass
