# Supplementary material for: A Sulfur-Crosslinked Biopolymeric Matrix for Controlled Urea Release Enhances Maize Growth and Reduces Nitrogen Losses
Source: Int J Mol Sci. 2026 Apr 27;27(9):3863. doi: 10.3390/ijms27093863 (PMC13164152; doi:10.3390/ijms27093863)
Supplement: Supplementary file 1 [file ijms-27-03863-s001.zip › ijms-4233252-supplementary.pdf]

Supplementary Materials: A Sulfur-Crosslinked Biopolymeric Matrix for Controlled Urea Release Enhances Maize Growth and Reduces Nitrogen Losses.

Ana Farioli, Pablo Cavallo, Diego Acevedo, and Edith Yslas.

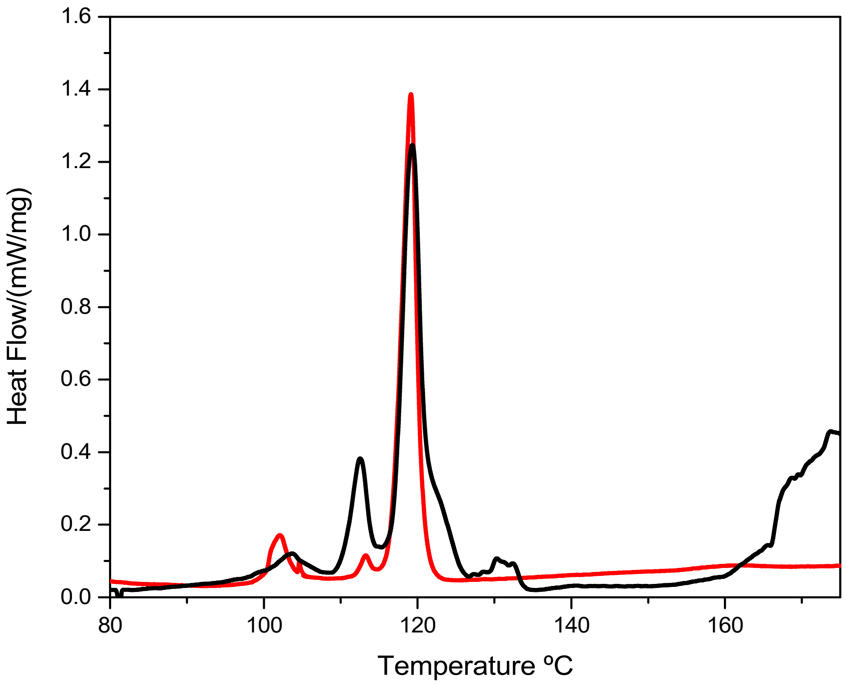

Figure S1: DSC thermograms of Bp-SF (red line), Bp-SF32U (black line), Temperature range: 80–175 °C.

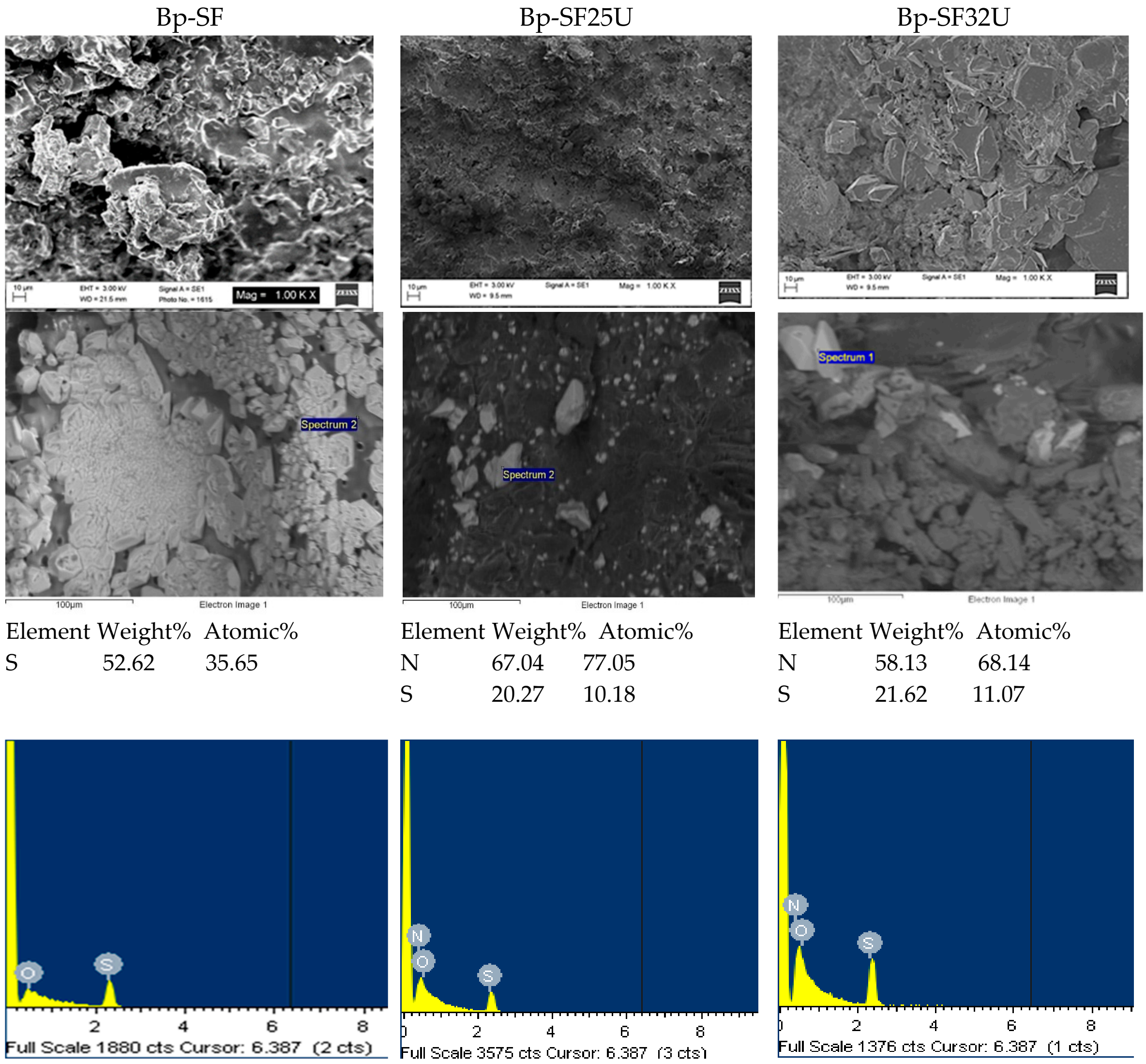

Figure S2. SEM images (1,000×), EDX spectra, and corresponding elemental composition (wt%) of Bp-SF, Bp-SF25U, and Bp-SF32U.
